# In Search of Healthy Ageing: A Microbiome-Based Precision Nutrition Approach for Type 2 Diabetes Prevention

**DOI:** 10.3390/nu17111877

**Published:** 2025-05-30

**Authors:** Adriana González, Asier Fullaondo, Adrian Odriozola

**Affiliations:** Department of Genetics, Physical Anthropology and Animal Physiology, University of the Basque Country (UPV/EHU), 48940 Bilbao, Spain; adriana.gonzalez@ehu.eus (A.G.); asier.fullaondo@ehu.eus (A.F.)

**Keywords:** type 2 diabetes (T2D), healthy ageing, T2D prevention, gut microbiota, microbiome-based precision nutrition, bioinformatics food recommendation system, overweight, 16S rRNA gene, MinION sequencing, NCBI taxonomic classification

## Abstract

**Background/Objectives**: Type 2 diabetes (T2D) is a leading cause of morbidity and mortality worldwide and in Spain, particularly in the elderly population, affecting healthy ageing. Nutritional strategies are key to its prevention. The gut microbiota is also implicated in T2D and can be modulated by nutrition. We hypothesize that precision nutrition through microbiota modulation may help prevent T2D. This article aims to (1) describe a gut microbiota bacterial profile associated with T2D prevention, (2) provide precision nutrition tools to optimize this profile, (3) analyze how overweight influences the microbiota composition and precision nutrition response, and (4) address the technical challenges of microbiome-based precision nutrition clinical implementation to prevent T2D. **Methods**: A review of gut microbiota associated with T2D prevention was conducted. 13 healthy Spanish participants over 62 with optimal blood glucose levels (7 normal weight and 6 overweight) underwent a 3-month precision nutrition intervention to optimize T2D-preventive gut microbiota using a bioinformatics food recommendation system, Phymofood (EP22382095). Fecal microbiota was analyzed pre- and post-intervention using full-length 16S rRNA gene amplification, MinION sequencing, and NCBI taxonomic classification. **Results**: 31 potentially preventive bacteria against T2D were selected. The intervention increased the relative abundance of beneficial genera (*Butyrivibrio* and *Faecalibacterium*) and species (*Eshraghiella crossota*, and *Faecalibacterium prausnitzii*). The overweight influenced microbiota composition and intervention response. **Conclusions**: A gut microbiota profile associated with T2D prevention was identified, and precision nutrition could increase the relative abundance of beneficial bacteria. Confounding factors such as overweight should be considered when designing microbiome-based precision nutrition interventions. These results contribute to a better understanding of the microbiota associated with T2D prevention and address technical challenges for clinical implementation in future healthy ageing strategies.

## 1. Introduction

Ageing is associated with an increased incidence of chronic diseases such as type 2 diabetes (T2D), which reduces life expectancy and quality in older people, posing a major challenge in ensuring healthy ageing. Specifically, each decade of suffering from T2D is estimated to result in losing 3–4 years of life [1]. According to the World Health Organization (WHO), the TD2 effect on years of life lost is particularly concerning in Spain, one of the countries with the highest life expectancy worldwide [2]. Although advances have been made in the prevention and treatment of T2D, it remains one of the main causes of morbidity and mortality in the world and Spain, especially in the elderly population [3].

Such is the importance of premature ageing that the WHO has proclaimed the Decade of Healthy Ageing (2021–2031) to enhance the quality of life in old age, and understanding the complexity of T2D is fundamental to meeting this challenge [3]. T2D accounts for about 90% of diabetes cases [4] and is a progressive metabolic pathology characterized by hyperglycemia distinguished by pancreatic β-cell malfunction and insulin resistance of peripheral tissues [5]. The progression of T2D involves chronic low-grade inflammation and can be associated with complications such as retinopathy, cardiovascular disease, or kidney failure [6]. The etiopathogenesis of T2D is influenced by genetic and environmental factors, highlighting unbalanced diets, overweight and obesity, sedentary lifestyles, and microbiota imbalance (dysbiosis). In this context, T2D is considered an epidemic and an emerging global public health problem. There is an urgent need to develop new strategies that act on these modifiable factors to improve the effective prevention of T2D and the development of associated complications [4].

In the last decade, the gut microbiota has emerged as a key factor in the etiopathogenesis of T2D. The gut microbiota is the community of microorganisms, consisting of bacteria, archaea, fungi, protozoa, and algae, that inhabit the host gut in a symbiotic manner. Intestinal microbiota dysbiosis is associated with T2D. Microbiota dysbiosis can increase intestinal permeability, promoting molecule translocation to the bloodstream and generating a strong immune response and a pro-inflammatory state [6]. Other mechanisms through which some gut microorganisms play a key role in T2D include metabolite production, glucose and lipid metabolism regulation, insulin sensitivity, and energy homeostasis [7].

Due to the role of the microbiota in T2D, it has become a therapeutic target of interest. There is growing scientific evidence for the modulation of the gut microbiota in preventing and treating T2D [4,8]. Different factors that can modulate gut microbiota have been described, such as nutrition, physical activity, circadian rhythms, and antibiotic exposure. Therefore, lifestyle interventions, especially nutrition, help to rebalance the gut microbiota [9].

Although diet is the main modulating factor of the microbiota, its impact on T2D cannot be analyzed without considering other factors that may affect its composition and response to nutritional interventions. For example, population group, age, blood sugar levels, and BMI are key factors in the relationship between microbiota and T2D [10,11]. In this sense, the population analyzed in this article is a Spanish group with optimal blood glucose levels and normal weight over 62 years of age, and a group of people with similar characteristics but who were overweight were analyzed to assess the impact of this potential confounder on their microbiota and response to interventions.

In this context, precision nutrition strategies can modulate the quantitative and qualitative composition of the gut microbiota, helping to prevent T2D and promote healthy ageing in increasingly long-lived populations such as the Spanish. Precision nutrition seeks personalization of dietary recommendations based on individual characteristics such as microbiota, health status, and dietary habits [12].

As the evidence on the relationship between microbiota and T2D grows, accurate taxonomic identification of bacteria, ideally to genus, species, and subspecies levels, is essential for understanding the specific pathophysiological role in T2D. In this sense, precision nutrition allows us to determine individual nutritional requirements for prebiotics (bacterial food), probiotics (beneficial bacteria), and postbiotics (metabolites produced by bacteria) to modulate the abundance of specific microbiota taxa previously associated with protective or detrimental potential in T2D [13,14,15].

However, certain challenges limit the effective clinical application of microbiome-based precision nutrition in T2D prevention. These limitations include the lack of reliable and clinically scalable identification of bacterial taxa associated with T2D, the high costs associated with microbiome analysis, the need to determine the relationship between the microbiota composition and food requirements, and the failure to consider possible confounding factors that mask the effects of precision nutrition on the microbiota [16,17]. Overcoming these limitations is key to applying microbiome-based precision nutrition to prevent and manage T2D and healthy ageing strategies.

First, it is essential to have tools for analyzing microbiota that provide robust and reproducible results to apply microbiome-based precision nutrition to prevent and manage T2D. In this regard, performing high-resolution bacterial taxonomic classification is essential to identify reliable and clinically scalable microbial biomarkers associated with T2D. It is crucial to consider methodological aspects such as the size of the amplicon to be sequenced and the selection of the database used for classification to achieve high taxonomic resolution.

Most studies analyzing microbiota and T2D use short specific regions (~200–400 bp) of the 16S ribosomal RNA (16SrRNA) gene, providing only accurate identification at the genus level [18]. However, amplifying and sequencing the whole gene (~1500 bp) allows for a higher precision in the microbiota taxonomic profile obtained [19]. Moreover, the taxonomic assignment of reads from the 16S rRNA gene is usually performed using SILVA, RDP, or Greengenes taxonomic databases. However, the NCBI taxonomy has more taxa and sequences and is constantly updated and curated, providing a more comprehensive classification. SILVA, RDP, or Greengenes databases have been reported to map well to NCBI but not vice versa. Therefore, NCBI is recommended for 16S studies because it classifies 16S rRNA gene sequence reads more effectively than the other taxonomies [20].

Regarding the cost-effectiveness of microbiome analysis, microbiome-precision nutrition is limited in clinical practice due to a lack of access to conventional sequencing technologies that are costly, time-consuming, and require infrastructure. In context, the MinION sequencing platform from Oxford Nanopore Technologies (ONT) is a revolution because of its potential for transferability into clinics due to its low cost, portability, and real-time sequencing capability [21,22].

To apply precision nutrition, selecting dietary components (food) to optimize the growth of the target bacteria is a crucial step. This process requires establishing the relationship between the composition of the microbiota and foods, directly or indirectly, through association with prebiotics and the amount of these prebiotics in specific foods, which is a real scientific challenge. The bioinformatics food recommendation system, Phymofood (EP4224486A1) [23], solved this challenge by developing the appropriate algorithm to quantify the influence on the state of the microbiota of a group of foods through knowledge of the prebiotics they include.

Finally, when applying microbiome-based precision nutrition, a key aspect to consider is that, as mentioned above, the microbiota is influenced by diet and other factors. Therefore, it is essential to consider variables such as BMI, which can act as confounding factors affecting the response to diet [16,24].

This article aims to (1) define a specific gut microbiota profile associated with T2D prevention, (2) provide precision nutrition tools to optimize this specific microbial profile and promote healthy ageing, (3) analyze if overweight affects the microbiota composition and precision nutrition response, and (4) overcome the main challenges for the implementation of microbiome-based precision nutrition in clinical practice to prevent and manage T2D.

For this purpose, the current scientific evidence related to T2D and gut microbiota was reviewed. A group of 7 healthy Spanish participants over 62 with optimal blood glucose levels and normal weight were recruited. Subsequently, a 3-month precision nutrition intervention using a bioinformatic food recommendation system was conducted to optimize the preventive gut microbiota of T2D and promote healthy ageing. Microbiota from feces samples was analyzed pre- and post-dietary intervention by complete sequencing of the 16S rRNA gene through the MinION sequencing platform and with the NCBI taxonomy database. In addition, an additional group of 6 overweight older people with optimal blood glucose levels were tested to see if this potential confounder affected microbiota composition and response to the intervention.

## 2. Materials and Methods

### 2.1. Literature Review

We conducted a literature search of the National Center of Biotechnology Institute’s PubMed and PubMed Central repositories using the title and abstract terms “type 2 diabetes” combined with “microbiota”, “bacteria”, “gut”, or “precision nutrition”, including articles published up to February 2025. Articles that analyze the relationship between T2D and commensal bacteria in the human gut were included. Studies with inconsistent results on the association between T2D and bacteria were excluded. Studies were also excluded if they dealt with bacteria previously associated with adverse health effects, if there were inconsistencies in the research, or if the evidence was insufficient. The Zotero software version 6.0.30 was used to manage the studies.

### 2.2. Study Design

A longitudinal study was carried out to compare the microbiota composition in healthy people over 62 years of age before and after 12 weeks of precision nutrition intervention for optimizing the T2D preventive microbiota.

### 2.3. Participants and Samples

A total of 14 pre–post nutrition intervention feces samples were obtained from 7 Spanish healthy participants, older than 62 years, with optimal blood glucose levels and normal weight. In addition, 12 pre–post nutrition intervention feces samples were analyzed from a second group of 6 healthy Spanish participants over 62 years of age with optimal blood glucose levels and overweight.

Feces samples were taken before and after 12 weeks of nutritional intervention by the DANASTOOL Sample Collection Microbiome Kit (Danagen-Bioted S.L., Barcelona, Spain) following the manufacturer’s indications. All samples were stored at −80 °C until DNA extraction.

The Medical Centre Ikigaia S.L. (Donostia-San Sebastián, Gipuzkoa, Basque Country, Spain) provided samples and data from the patients included in this study. They were processed according to the standard operating procedures approved by the Research Ethics Committee of Gipuzkoa Healthcare Area (Basque Health Service (Osakidetza)) (LMJ-BIZ-2021-01). All individuals gave written informed consent.

The participants were adequately informed about risks and benefits, according to the EU General Data Protection Regulation (GDPR), the Regulation (EU) 2016/679 of the European Parliament and of the Council of 27 April 2016 on the protection of natural persons about the processing of personal data and the free movement of such data, and repealing Directive 95/46/EC (General Data Protection Regulation), OJ 2016 L 119/1.

Anthropometric weight and height measurements were taken to establish body mass index (BMI). The WHO criteria for BMI values were followed. Thus, subjects with a BMI ≤ 24.9 kg/m^2^ were classified as normal weight, whereas subjects with a BMI ≥ 25 kg/m^2^ and ≤29.9 kg/m^2^ were classified as overweight.

Subjects with normal fasting blood glucose levels (FG) were selected based on the Basque Health Service (Osakidetza) criteria to which this population belongs. Osakidetza uses a diagnostic and screening algorithm for T2D based on the Clinical Practice Guidelines (CPG) of the Spanish National Health System, aligned with the WHO and the International Diabetes Federation (IDF), also considering the American Diabetes Association (ADA) [25].

According to this CPG, normal FG values are defined as <110 mg/dL or <100 mg/dL, according to the most restrictive ADA criteria [25]. In this study, an even more restricted threshold was set by choosing only those individuals with FG values < 95 mg/dL to ensure an optimal glycemic profile in the sample.

### 2.4. Precision Nutrition Intervention

The precision nutrition intervention lasted 12 weeks and was supervised by a nutrition professional. It consisted of a normocaloric and balanced diet, following the guidelines established by the European Food Safety Authority (EFSA) for adults, with an energy intake of 45–60% carbohydrates, 20–35% total fat (with less than 10% of saturated fat), and 10–20% protein [26]. Diet included an adequate amount of micronutrients and was enriched in nutrients based on the food recommendation system Phymofood developed and patented by our research group (EP4224486A1) [23], owned by the University of the Basque Country (UPV) and the University of Cantabria (UC).

### 2.5. DNA Extraction and Quantitation

DNA was extracted using the DANAGENE Microbiome Fecal DNA kit (Danagen-Bioted S.L., Barcelona, Spain). DNA was quantified by fluorometry (Qubit 2.0, Life Technologies, Carlsbad, CA, USA, Thermo Fisher Scientific, Waltham, MA, USA) using HS dsDNA Assay (Thermo Fisher Scientific, Waltham, MA, USA) and by spectrophotometry (NanoDrop 2000c, Thermo Fisher Scientific, Waltham, MA, USA). Negative controls were used for DNA extraction.

### 2.6. PCR Amplification

PCR amplification of full-length 16S rRNA genes (~1500 pb) was performed using the 16S Barcoding Kit (SQK-RAB204; Oxford Nanopore Technologies, Oxford, UK) with the 27F/1492R primer set and LongAmp™ Taq 2× Master Mix (New England Biolabs, Ipswich, MA, USA). This process was carried out on an Applied Biosystems Veriti™ Thermal Cycler (Thermo Fischer Scientific, Waltham, MA, USA) with the following PCR conditions recommended by the manufacturer: initial denaturation at 95 °C for 3 min, 25 cycles of 95 °C for 20 s, 55 °C for 30 s, and 65 °C for 2 min, followed by a final extension at 65 °C for 5 min. Negative controls were used in PCR amplification.

### 2.7. Library Preparation and Amplicon Sequencing

PCR products were purified using Clean NGS CleanNGS (CleanNA, Waddinxveen, The Netherlands). Then, each eluate was quantified by fluorometry (Qubit 2.0, Life Technologies, Carlsbad, CA, USA, Thermo Fisher Scientific, Waltham, MA, USA) using HS dsDNA Assay (Thermo Fisher Scientific, Waltham, MA, USA) for calculating DNA input for library preparation.

A total of 100 ng DNA per sample was used for barcoded library preparation. Multiplex sequencing was performed using a MinION Mk1B nanopore sequencing device (MIN-101B), Flonge Adapter (ADP-FLG001), and Flongle Flow Cell (FLO-FLG001) according to the manufacturer’s instructions (Oxford Nanopore Technologies, Oxford, UK).

### 2.8. Data Acquisition and Sequencing Data Analysis

MINKNOW UI software version 23.04.3 (Oxford Nanopore Technologies, Oxford, UK) was used for data acquisition and base-calling, converting sequence reads (i.e., FAST5 data) into FASTQ files using the Guppy pipeline version 6.5.7 (Oxford Nanopore Technologies, Oxford, UK).

Taxonomic classification was performed using the Barcoding workflow in the analysis platform EPI2ME of Metrichor Ltd. (Oxford Nanopore Technologies, Oxford, UK). To that aim, FASTQ files were uploaded to the 16S workflow of the EPI2ME desktop agent, where real-time classification was carried out using the NCBI 16S rRNA gene blast database. A quality filter for reads of Q-score ≥ 9 was applied. Data acquisition and base-calling in real-time using the MinION were carried out over 48 hours. The results were processed by in-house software to avoid biases in the relative quantification of taxa. For this purpose, reads were converted into relative abundance considering each taxon’s estimated 16S gene copy number (GCN) according to the rrnDB database version 5.9 [27].

### 2.9. Bioinformatics Analysis

Different α-diversity analyses were performed. On the one hand, community richness was calculated using the number of observed Operational Taxonomic Units (OTUs) and the Chao1 index. On the other hand, diversity and evenness were estimated through the Shannon–Weaver index [28] and the Simpson index [29]. The α-diversity analysis was performed and visualized using box plots in Python 3.11. For β-diversity analysis, the following two metrics were calculated from OTUs relative abundance: Bray–Curtis dissimilarity and Jaccard index. The β-diversity results were visualized using Principal coordinates analysis (PCoA) in PAST 4.13. A bioinformatics analysis was carried out to establish specific microbiota compositional differences between groups using GraphPad 8.0. Differential abundance analysis results were visualized using stacking maps and violin plots in SRPlot [30].

### 2.10. Statistical Analysis

A Mann–Whitney U test was applied to evaluate differences in α-diversity metrics means between paired groups. A Wilcoxon signed-rank test was used to analyze differences in α-diversity means between unpaired groups. A *p*-value < 0.05 was considered statistically significant.

Analysis of Similarities (ANOSIM) and Permutational Multivariate Analysis of Variance (PERMANOVA) tests were used to evaluate differences in β-diversity metrics between groups in PAST 4.13.

OTUs relative abundance differences between groups were evaluated using multiple *t*-tests in GraphPad Prism 8.0. The Benjamini, Krieger, and Yekutieli method was applied to control the False Discovery Rate (FDR) and consider multiple comparisons. FDR-adjusted *p*-values < 0.01 were considered statistically significant.

## 3. Results

### 3.1. Outcomes of Study Selection

The search resulted in 2920 articles. Of these, 628 articles related to T2D and human gut commensal bacteria were selected to identify the most significant bacteria at the genus and species levels. Among these, 52 articles were selected that consistently discussed the association of bacteria with T2D. This preliminary selection identified bacteria previously associated with adverse health effects, and the corresponding articles were discarded. In the end, 32 articles concerning gut commensal bacteria associated with T2D prevention were used to elaborate Table 1.

### 3.2. Target Bacteria Associated with T2D Prevention

For potential T2D-preventive bacteria identification, 48 were preliminarily selected because they were detected in reduced relative abundance in TD2 subjects in comparison to controls in previous studies, they were associated with low blood glucose levels, and there was functional evidence regarding their potential preventive role in T2D development. 17 of those bacteria were discarded because they were related to adverse health effects in other studies, inconsistencies in the scientific literature or insufficient evidence. Finally, 31 bacteria were selected and grouped in Table 1, according to the available scientific evidence supporting their potentially preventive role in T2D. The target bacteria selection and bacteria discard criteria are explained in detail below.

#### 3.2.1. Target Bacteria Selection

Selecting target bacteria is fundamental to developing a microbiome-based precision nutrition strategy. In this paper, after identifying 48 bacteria that were reduced in T2D, only 31 were selected as potentially preventive (Table 1).

*Akkermansia muciniphila* plays a beneficial role in T2D by modulating inflammation, decreasing intestinal permeability, influencing glucose metabolism, and promoting fatty acid oxidation in adipose tissue [7,31,32].

*Anaerobutyricum soehngenii* contributes positively, improving peripheral insulin resistance and lowering the risk of developing T2D [33,34,35].

In T2D, a decrease in the bacteria with anti-inflammatory properties has been found, such as *Anaerostipes*, *Coprococcus*, *Lachnospira* and *Phascolarctobacterium*, because of their ability to produce short-chain fatty acids (SCFAs), such as butyrate [36]. Reduction in butyrate-producing bacteria in patients with T2D has been associated with insulin resistance [36,37,38,39,40]. In addition, butyrate can also potentially reduce intestinal permeability, one of the mechanisms associated with T2D [41].

Other butyrate-producing bacteria reduced in T2D include *Butyrivibrio fibrisolvens* [42], *Roseburia inulinivorans* [43,44], *Roseburia intestinalis* [44,45], together with *Faecalibacterium prausnitzii* [44,46,47,48], belonging to the *Clostridium coccoides* (XIVa) and *Clostridium leptum* (IV) clusters, respectively. These bacterial clusters, which are the most dominant in butyrate production in the gut and contribute significantly to its generation [49], are decreased in patients with T2D [50,51,52,53].

Furthermore, research suggests that *Bacteroides* species positively affect glucose metabolism in humans and experimental animals [7,54]. In particular, *Phocaeicola dorei* (previously known as *Bacteroides dorei*) is considered potentially beneficial due to its immunomodulatory capacity correlated with glycemic reduction in patients with T2D, as well as its ability to decrease intestinal permeability, lipopolysaccharide production, and endotoxemia in murine models [43,54,55].

Also, *Bacteroides fragilis* is beneficial for T2D because it produces anti-inflammatory cytokines that improve glucose metabolism and protect against insulin resistance [7,54]. For its part, *Bacteroides thetaiotaomicron* can exert beneficial effects on T2D by reducing pro-inflammatory cytokines. T2D is associated with increased levels of pro-inflammatory cytokines, chemokines, and inflammatory proteins [7,54].

*Bifidobacterium* is a genus with a protective role against T2D, which is consistently supported by the scientific literature by containing potentially protective bacteria [7,54].

*Blautia wexlerae* has beneficial effects that reduce the risk of T2D. This bacterium produces several unique metabolites capable of altering energy metabolism, producing anti-inflammatory effects, and changing the composition of gut microbiota bacteria and SCFAs [56,57].

Finally, *Eshraghiella crossota* (previously known as *Butyrivibrio crossotus*) is a bacterium with protective potential against T2D. The reduction in this bacterium, among others, has been associated with the reduced bacterial capacity of branched-chain amino acid (BCAA) uptake. The increased ratio between the gut microbiota for BCAA biosynthesis and uptake contributes to increased serum BCAA concentrations associated with insulin resistance [58,59].

In the coming years, advances are likely to continue in the area, so the list of T2D preventive-associated gut microbiota bacteria in Table 1 remains open to including new bacteria.

**Table 1:** T2D preventive-associated gut microbiota bacteria were reduced in T2D compared to the healthy group.

Bacteria	TaxID	References
*Akkermansia muciniphila*	239935	[7,31,32]
*Anaerobutyricum soehngenii*	105843	[33,34,35]
*Anaerostipes*	207244	[36,37,38,39,40]
*Bacteroides*	816	[7]
*Bacteroides fragilis*	817	[7,54]
*Bacteroides intestinalis*	329854	[7,45,54]
*Bacteroides ovatus*	28116	[54]
*Bacteroides stercoris*	46506	[7,54]
*Bacteroides thetaiotaomicron*	818	[54]
*Bacteroides uniformis*	820	[54]
*Bifidobacterium*	1678	[7,54]
*Bifidobacterium adolescentis*	1680	[54,60,61]
*Bifidobacterium animalis*	28025	[54]
*Bifidobacterium bifidum*	1681	[54,60,61]
*Bifidobacterium breve*	1685	[54,62]
*Bifidobacterium dentium*	1689	[54,60,61]
*Bifidobacterium longum*	216816	[54,60,61]
*Bifidobacterium pseudocatenulatum*	28026	[54,60,61]
*Blautia wexlerae*	418240	[56,57]
*Butyrivibrio fibrisolvens*	831	[42]
*Clostridium coccoides* cluster	1532	[51,52]
*Clostridium leptum* cluster	1535	[50,51,52,53]
*Coprococcus*	33042	[36,39]
*Eshraghiella crossota* (*Butyrivibrio crossotus*)	45851	[58,59]
*Faecalibacterium*	216851	[7,38,63]
*Faecalibacterium prausnitzii*	853	[44,46,47,48]
*Lachnospira*	28050	[36,40,64]
*Phascolarctobacterium*	33024	[36]
*Phocaeicola dorei* (*Bacteroides dorei*)	357276	[43,54]
*Roseburia inulinivorans*	360807	[43,44]
*Roseburia intestinalis*	166486	[44,45]

The taxonomic name refers to the current name in the NCBI Taxonomy Browser. TaxId refers to the taxonomy identifier, numeric, unique, and stable, associated with each NCBI Taxonomy entry (https://www.ncbi.nlm.nih.gov/Taxonomy/Browser/wwwtax.cgi; accessed on 14 February 2025).

#### 3.2.2. Target Bacteria Discard

From the final T2D preventive-associated gut microbiota list (Table 1), 17 bacteria were discarded due to their association with adverse health effects, discrepancies in the investigations, or insufficient evidence.

*Haemophilus* and *Comamonas*, although reported to be decreased in T2D, are not included because they are considered possibly pathogenic bacteria. Similarly, it has been suggested that *Parabacteroides merdae* may have a protective role against T2D. However, more research is needed as it is widely distributed in the microbiome of hypertensive patients and is an opportunistic pathogen [53]. Although *Sutterella* has been reported as reduced in T2D, it is not included because it is a pro-inflammatory bacterium [36,44,45].

*Bacteroides vulgatus* and *B. xylanisolvens* have been detected in lower amounts in the T2D group compared to the control in several studies [7,50,54]. However, it has also been associated with the development of T2D [65].

*Blautia* has been reported in several studies to be decreased in patients with T2D [36,38,56], while in others to be increased [7,66,67]. This increase has been associated with metformin administration, but the beneficial effects of *Blautia* on T2D are still controversial [68].

*Butyricicoccus* is a genus known for its anti-inflammatory properties, which have been positively correlated with glycemic reduction [43]. However, *Butyricicoccus* has also been identified as one of the unique nuclei of the microbiome associated with T2D. The authors suggested its presence might be related to increased pathogens [36].

*Collinsella aerofaciens* has been reported at a significantly lower level in the T2D group compared to the control in some studies [69,70], while in others, it was increased [71,72,73].

*Dorea*, a butyrate producer, is reduced in some T2D patients [64] but increased in others [74]. It has also been associated with a modestly lower risk of T2D [38].

*Roseburia* is a butyrate-producing genus consistently negatively associated with T2D [7,36]. However, it is not included because a recent Mendelian randomization study identified causal relationships with T2D and suggested that *Roseburia* may have an anti-protective effect on this disease [75].

*Lactobacillus* is frequently detected and reported in studies investigating T2D, but discrepant results are shown [7].

*Lachnoclostridium* is not included because it has been associated in one study with glycemic reduction [43], while another paper suggests that it might be causally associated with T2D [75].

*Parabacteroides distasonis* is not included because the evidence of its impact on T2D is limited and controversial. This bacterium has been proposed as a potential taxonomic biomarker of T2D [54]. However, it is difficult to classify it as a beneficial commensal or pathogenic bacterium because it can not only prevent metabolic dysfunctions but also negatively affect diabetes [76].

*Subdoligranulum* is an opportunistic butyrate-producing pathogen whose physiological role is unknown [77]. This bacterium is decreased in the T2D group compared to the control group in some studies [78] and in others increased [46,54,79]. In addition, *Subdoligranulum variabile* is one of the most enriched bacteria in T2D patients under treatment with the most prescribed anti-diabetic drug, metformin [54].

*Christensenella minuta* is a potentially beneficial bacterium for improving T2D but is not yet included due to a lack of human studies [80,81].

Some bacteria, such as *Turicibacter*, have been reported to be decreased in T2D, but their role in T2D is not yet known [36,40].

Therefore, because these bacteria are potential pathogens or present inconsistent or insufficient evidence, they are not currently included as target bacteria. However, we open the possibility of reconsidering these bacteria in future reviews.

### 3.3. Participant Baseline Characteristics

Table 2 shows the baseline characteristics of the participants. The normal weight group had a mean age of 67 ± 2 years, with a BMI of 21 ± 1.4 kg/m^2^. There were 57.1% women and 42.9% men. Mean blood sugar levels were 85 ± 5.8 mg/dL. The percentage of participants that reported personal food preferences was 28.6%. Participants reported a mean of 9 ± 7.7 h per week of physical exercise, practicing different types of activities as follows: walking and yoga (14.3%); running, cycling, and swimming (57.1%); or weights (28.6%).

The mean age of the overweight group was 64 ± 1.3 years, with a BMI of 27 ± 1.7 kg/m^2^. Among them, 66.7% were women and 33.3% were men. Participants showed mean blood sugar levels of 87 ± 5.3 mg/dL. The percentage of participants that reported personal food preferences was 33.3%. Regarding physical exercise, a mean of 3 ± 3.2 h per week was reported, practicing different activities, such as walking and yoga (50%) or running, cycling, and swimming (50%).

### 3.4. Microbiome-Based Precision Nutrition Intervention

The most significant gut microbiota bacteria associated with T2D prevention (target bacteria) were selected (Table 1). The Phymofood bioinformatics tool (EP4224486A1) [23] was used as a precision nutrition strategy to select the foods that promote optimal growth of the target bacteria for each study participant.

In general terms, Phymofood employs an algorithm to quantify the influence on the microbiota status of a food group through the knowledge of the prebiotics they include. The selection process of foods is based on the percentage of target bacteria theoretically fed by each diet component and the specific prebiotics of the diet component. The result is an individualized ranking of foods according to the degree to which they can support an improvement in the composition of the target microbiota in each case. Although the algorithms and the complete procedure are detailed in patent EP4224486A1, the main features and steps are detailed below [23,82].

First, the contribution of each food to the growth of each target bacterium is assessed on a scale of 0 to 1. A maximum value (1) is assigned if there is direct scientific evidence of an association between the specific food and the growth of target bacteria. If no direct association is known, values are assigned to each food depending on whether or not the prebiotics each food contains are target bacterium prebiotics. In this case, a maximum value of 1 is given if all prebiotics contained in the food are target bacterium prebiotics, a minimum value of 0 if none are, and an intermediate value if only some are, based on a harmonic series. Then, a vector that records the values assigned previously for all target bacteria is created for each food. Finally, the foods are ranked according to the sum of all values, with the first contributing most to the growth of bacteria associated with T2D prevention [23,82].

The Phymofood system evaluated a set of 99 foods and 18 prebiotics, including xylooligosaccharides, resistant starch, fructooligosaccharides, inulin, pectin, resveratrol, quercetin, raffinose, arabinoxylans, arabinoxylan-oligosaccharides, arabinogalactans, galactooligosaccharides, beta-glucans, lignans, ellagitannins, curcumin, anthocyanins, and isoflavones.

The process carried out by the Phymofood system allowed the design of a precision nutrition diet based on the gut microbiota composition in each subject to optimize the growth of T2D preventive bacteria.

### 3.5. α- and β-Diversity

#### 3.5.1. α-Diversity of Microbiota Pre-Dietary Compared to the Post-Dietary Intervention of Subjects with Normal Weight

**Figure 1:**
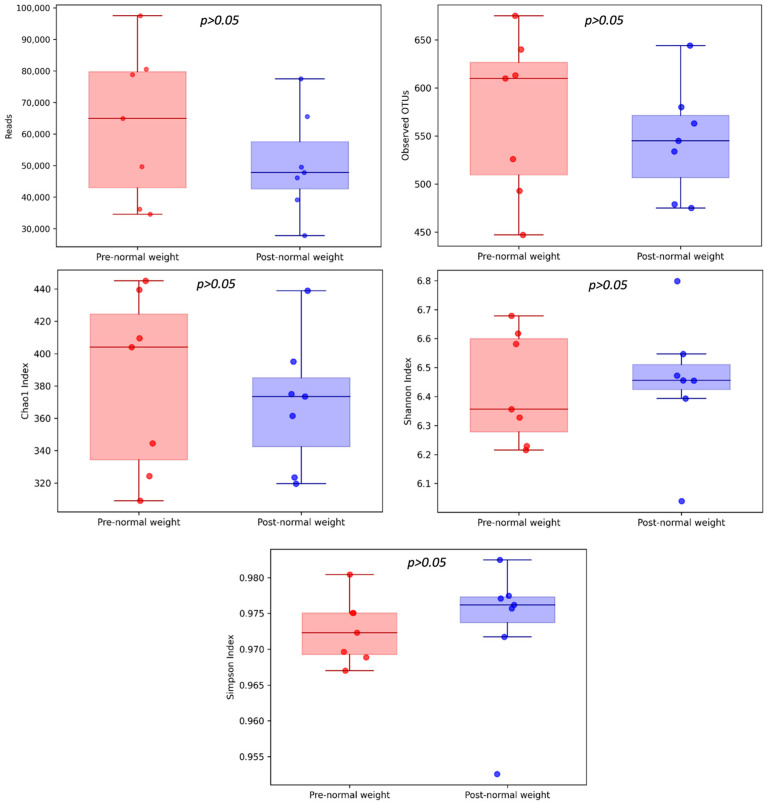
α-diversity of pre- and post-dietary intervention from normal weight samples. Comparison of community richness (number of OTUs, Chao1 index) and diversity and evenness (Shannon and Simpson indexes).

We analyzed the microbiota composition of pre-dietary compared to post-dietary intervention samples from 7 healthy subjects with normal weight over 62 years old. The average reads per sample for the pre- and post-intervention samples (mean value ± standard error) were 63,152 ± 22,238 and 50,471 ± 15,272, respectively (*p* = 0.578) (Figure 1). Overall, 1266 different OTUs were identified at a 97% similarity threshold.

We observed a trend towards higher community richness for the Chao1 index and diversity and evenness (Shannon and Simpson indexes) in post-dietary compared to pre-dietary intervention samples, suggesting a greater microbial diversity in this group. The number of OTUs was slightly lower in the post-intervention compared to the pre-intervention group (Figure 1).

**Figure 2:**
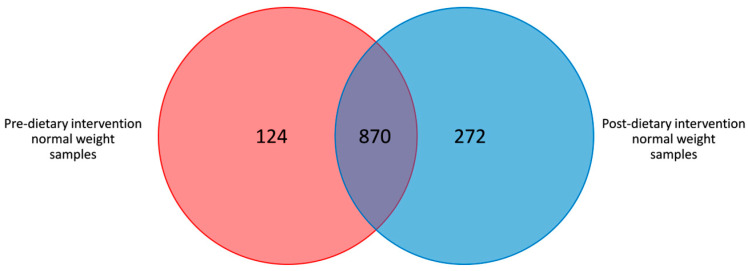
Venn diagram of the shared OTUs among pre- and post-dietary intervention from normal weight samples.

In total, 1266 OTUs were identified, with 870 OTUs shared among pre- and post-intervention samples (Figure 2). The average number of OTUs in the normal weight samples pre- and post-intervention was 572 ± 78 and 545 ± 54, respectively (*p* = 0.688).

However, α-diversity comparison between the pre- and post-intervention groups did not report statistically significant differences among the metrics analyzed as follows: Chao1 (382.26 ± 51.53 and 369.59 ± 38.2, respectively; *p* = 0.937), Shannon index (6.43 ± 0.18 and 6.45 ± 0.21, respectively; *p* = 0.937), and Simpson indexes (0.97 ± 0.004 and 0.97 ± 0.009, respectively; *p* = 0.578) (Figure 1).

#### 3.5.2. β-Diversity of Microbiota in the Pre-Dietary Compared to the Post-Dietary Intervention of Subjects with Normal Weight

**Figure 3:**
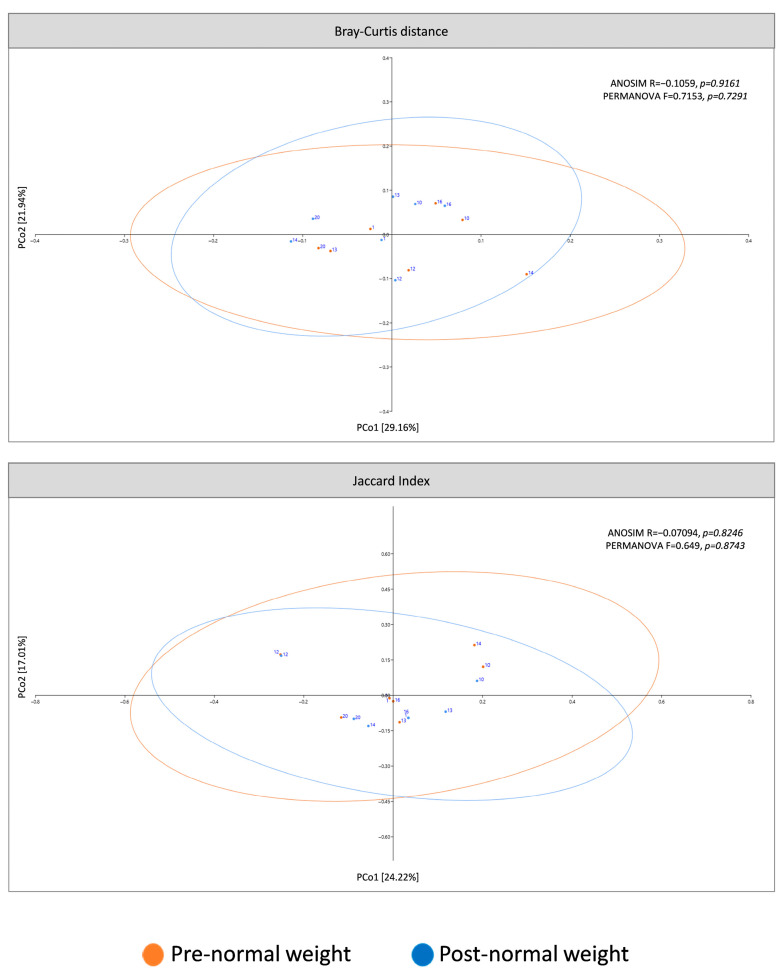
β-diversity of pre-dietary compared to post-dietary intervention from normal weight samples. The principal coordinates analysis (PCoA) plots were based on the following two metrics: Bray–Curtis dissimilarity and Jaccard index (ellipses represent 95% confidence intervals).

The present study analyzed β-diversity between pre- and post-dietary intervention samples using PCoA of two metrics (Bray–Curtis and Jaccard) and ANOSIM and PERMANOVA analysis (Figure 3). Analysis revealed that microbiota profiles did not differ between pre- and post-intervention groups.

### 3.6. Specific Microbiota Compositional Differences Between Pre- and Post-Dietary Intervention of Subjects with Normal Weight

Although the pre-dietary compared to post-dietary intervention groups did not show significant community-level differences, significant differences in the relative abundance of specific taxa were found between the two groups (Table 3 and Table 4).

**Figure 4:**
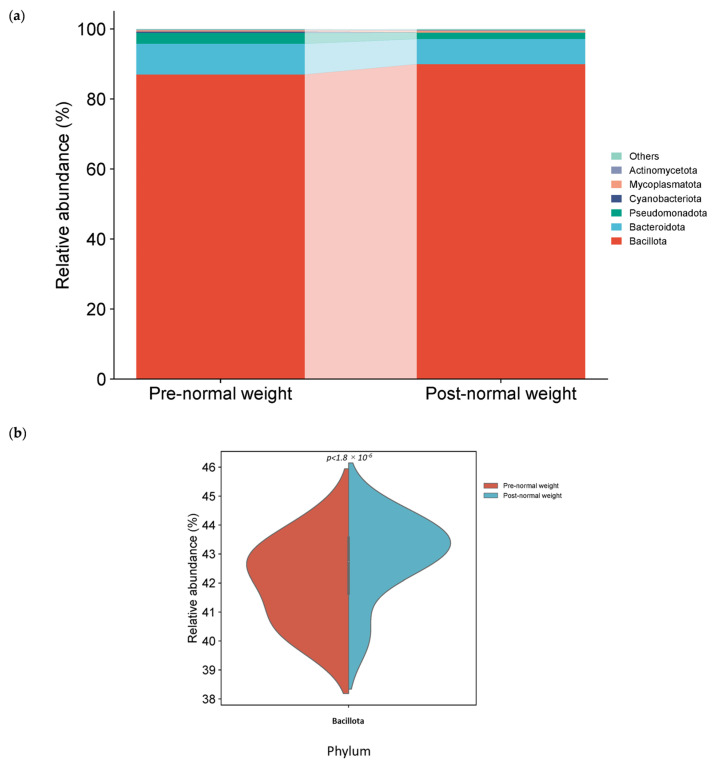
Different bacterial distribution in pre-dietary compared to post-dietary intervention in normal weight samples at the phylum level. (**a**) Stacked bar plots showing the distribution of bacterial taxa at the phylum level. (**b**) Violin plot illustrating the relative abundance of the phylum showing significant differences between groups pre- and post-intervention: Bacillota.

The phyla Bacillota, Bacteroidota, and Pseudomonadota (formerly Proteobacteria), regular members of the human gut microbiota, formed more than 98% of the bacterial phyla in pre- and post-dietary intervention in normal weight samples. Among them, the phylum Bacillota was the most abundant in both groups, with a relative abundance of more than 85% (Figure 4a). Relative abundances at the phylum level were compared pre- and post-intervention in normal weight samples, and a significant increase in the phylum Bacillota was observed in the post-intervention group (Table 3 and Figure 4b).

**Figure 5:**
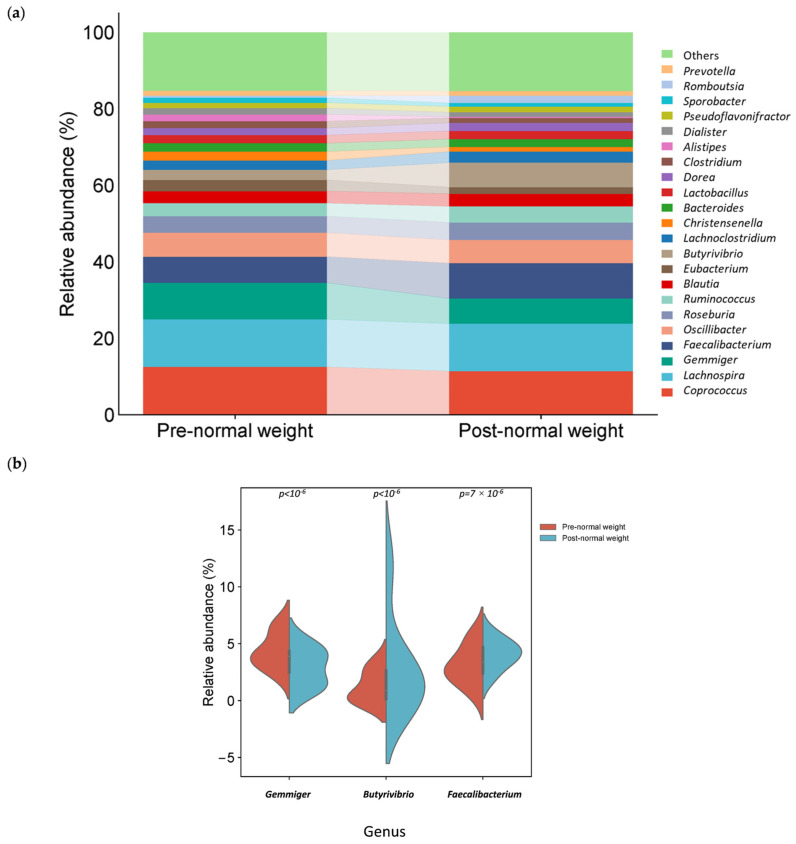
Different bacterial distribution in pre-dietary compared to post-dietary intervention in normal weight samples at the genus level. (**a**) Stacked bar plots showing the distribution of bacterial taxa at the genus level. (**b**) Violin plot illustrating the relative abundance of the genus showing significant differences between groups pre- and post-intervention: *Gemmiger*, *Butyrivibrio*, and *Faecalibacterium*.

*Coprococcus*, *Lachnospira*, *Gemmiger*, *Faecalibacterium*, and *Oscillibacter* were the fifth most abundant genus pre-dietary compared to post-dietary intervention in normal weight samples. Interestingly, among the 20 most abundant genera in pre- and post-intervention samples, 18 were common, while *Alistipes* and *Sporobacter* were also found in the pre-intervention group, whereas *Rombutsia* and *Prevotella* were in the post-intervention group (Figure 5a). The relative abundance of one genus, *Gemmiger*, was significantly higher in pre-intervention samples (Table 3 and Figure 5b). In contrast, the genera *Butyrivibrio* and *Faecalibacterium* were significantly higher in post-intervention samples (Table 4 and Figure 5b).

**Figure 6:**
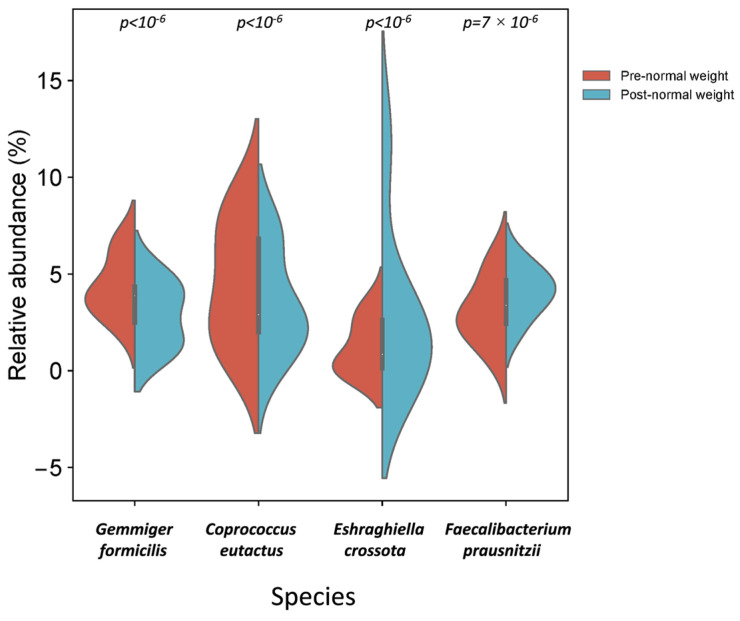
Different bacterial distribution among pre- and post-dietary intervention normal weight samples at the species level. Violin plot representing the relative abundance of the species significantly different between pre- and post-intervention groups: *Gemmiger formicilis*, *Coprococcus eutactus*, *Eshraghiella crossota*, and *Faecalibacterium prausnitzii*.

In the pre-intervention group, species assigned to *Gemmiger formicilis* and *Coprococcus eutactus* had significantly higher relative abundances than the post-intervention group (Table 3 and Figure 6). In contrast, *Eshraghiella crossota* and *Faecalibacterium prausnitzii* were significantly more abundant in the post-intervention group (Table 4 and Figure 6).

### 3.7. Type 2 Diabetes-Associated Microbiota Differences Related to Overweight

Due to the influence of obesity on the risk of developing T2D, we examined possible differences in gut microbiota profiles in stool samples based on their BMI values.

Similarly, there were no significant differences in normal weight samples compared to overweight samples in any of the metrics analyzed for the α-diversity in either the pre-dietary intervention group (Supplementary Figure S1) or the post-dietary intervention group (Supplementary Figure S2) or for the β-diversity tests (Supplementary Table S1).

At the taxonomic level, there were significant differences between the BMI groups. Our results indicated that pre-dietary intervention, microbiota from normal weight, compared to the overweight samples, was characterized by an enrichment of the phylum Bacillota, class Clostridia, and order Eubacteriales. In contrast, a decrease was observed in phylum Bacteroidota (Table 5).

After dietary intervention, the microbiota from normal weight, compared to the overweight samples, was characterized by a preponderance in the family Lachnospiraceae, genus *Coprococcus* and *Butyrivibrio*, and species *E. crossota* and *C. eutactus*. In contrast, the microbiota of normal weight was significantly decreased with the family Oscillospiraceae, genus *Oscillibacter*, and species *Eubacterium siraeum* and *Oscillibacter valericigenes* (Table 6).

Finally, there were no significant differences in the α-diversity metrics or β-diversity tests between the pre- and post-dietary intervention overweight samples (Supplementary Figures S3 and S4). However, significant differences were observed in the relative abundance of certain bacterial taxa among the pre- and post-dietary intervention overweight groups, with a significantly higher relative abundance of the genera *Coprococcus* pre-intervention (Table 7).

## 4. Discussion

In the present research, we analyzed the microbiota composition of the pre- and post-dietary intervention from 7 healthy Spanish participants older than 62, with optimal blood glucose levels and normal weight. Microbiota analysis was performed by amplifying the 16S rRNA gene using the MinION sequencing platform and NCBI taxonomic classification. This methodology allowed the identification of significant variations in the relative abundance of bacteria taxa between normal weight samples before and after dietary intervention, consistent with previous studies, even at more specific taxonomic levels, such as species.

To identify potentially T2D-preventing bacteria, 48 were preliminarily selected based on a significant relative abundance reduction in T2D subjects compared to the healthy group in previous research, low blood glucose levels, and functional evidence regarding possible preventive effects in T2D development. However, 17 of these bacteria were discarded due to their pathogenic potential, inconsistencies in the findings reported in the scientific literature, or insufficient evidence. Finally, 31 bacteria were selected and included in Table 1, according to the available scientific evidence on their association with T2D prevention. The selection of bacteria associated with the prevention of T2D (Table 1) could improve the scientific basis for developing and implementing microbiome-based precision nutrition strategies that contribute to preventing T2D and its complications.

There were no differences at the community level when analyzing α- and β-diversity between pre- and post-dietary intervention normal weight samples (Figure 1 and Figure 2). However, no changes in these indices were expected due to the intervention since the key approach for evaluating precision nutrition effects lies in analyzing the relative abundances of target taxa related to T2D prevention. In this regard, the differential abundance analysis revealed that there were taxa with significant variations between the two groups (Table 3 and Table 4).

The relative abundance of genus *Gemmiger* (*p* < 10^−6^) and species *G. formicilis* (*p* = 2 × 10^−6^) and *C. eutactus* (*p* = 1.9 × 10^−5^) was significantly increased in pre-dietary intervention normal weight samples (Table 3).

The relative abundance of the potentially detrimental bacteria *Gemmiger formicilis* was significantly reduced in normal weight samples after the dietary intervention. *G. formicilis* has been previously associated with Crohn’s disease, where it was significantly increased and associated with the recurrence of inflammatory lesions. It has been suggested that this bacterium could play a pro-inflammatory role, intensifying inflammation and worsening the disease symptoms [83,84,85].

Unexpectedly, the relative abundance of the butyrate-producing bacteria *C. eutactus* was significantly reduced in normal weight samples after the dietary intervention. *C. eutactus* was associated with insulin sensitivity [86]. *C. eutactus* was found depleted in subjects with prediabetes, an intermediate stage in the development of T2D [57]. An increase in beneficial bacteria has been observed in T2D, which has been suggested to counteract the increase in pathogens [36]. Perhaps the reduction in *C. eutactus* is due to a reorganization of the community. Increasing knowledge about the interactions between beneficial taxa, such as *C. eutactus*, and pathogens could improve microbiome-based precision nutrition interventions for T2D prevention.

Moreover, the relative abundance of phyla Bacillota (*p* = 1.5 × 10^−3^), genus *Butyrivibrio* (*p* < 10^−6^), and *Faecalibacterium* (*p* = 7.2 × 10^−4^) and species *E. crossota* (*p* < 10^−6^) and *F. prausnitzii* (*p* = 7.2 × 10^−4^) was significantly reduced in post-dietary intervention normal weight samples (Table 4).

The relative abundance of *E. crossota*, a butyrate-producing bacterium, was significantly increased in normal weight samples after the dietary intervention. It has been recently found that *E. crossota* was depleted in patients with T2D [59]. It has been suggested that an increased ratio between the biosynthesis and uptake of BCAAs by the gut microbiome promotes an increase in BCAA serum concentrations, which is associated with insulin resistance, a precursor state to T2D. In this context, a reduced capacity for bacterial uptake of BCAAs has been associated with a reduced abundance of various species, including *E. crossota* [58].

The genus *Faecalibacterium* and species *F. prausnitzii*, a butyrate-producing bacteria, were significantly increased in normal weight samples after the dietary intervention. A consistent negative association between the genus *Faecalibacterium* and the species *F. prausnitzii* has been reported with T2D [7]. Another species of this genus, *Faecalibacterium cf.*, has been associated with diabetes remission after bariatric surgery [60].

The perspective and results obtained in this paper align with previous research exploring the effect of precision nutrition on the gut microbiota in the context of T2D. Recently, the importance of specific prebiotics in increasing the number of beneficial bacteria for T2D has been highlighted [13]. Specifically, it has been reported that a diet rich in resistant starch can increase *Faecalibacterium prausnitzii* in individuals with low insulin sensitivity [87]. In addition, probiotic-based nutritional interventions have also been used to maintain glucose homeostasis in patients with T2D [15].

Although the results of our intervention are preliminary, they are aligned with the existing literature and suggest that the microbiome-based precision nutrition tool improves the relative abundance of bacteria associated with T2D prevention. In this context, the review and discussion of knowledge in this article may serve as a basis for using bioinformatics tools, such as the Phymofood food recommendation system (EP4224486A1) [23], as a precision nutrition strategy to personalize the diet to optimize the growth of target bacteria of the human gut microbiota for their preventive role against T2D.

In addition, we present the results of our research on gut microbiota profiles in healthy individuals over 62 years of age, comparing those who were normal weight and overweight before and after the precision nutrition intervention. Although there were no significant differences in the analysis of α- and β-diversity, we identified the relative abundance of specific taxa as significantly different (Table 6).

Interestingly, post-dietary intervention, samples of normal weight subjects showed an increased abundance of the butyrate-producer species, *E. crossota* and *C. eutactus* [88].

In contrast, the samples from overweight subjects showed an enrichment of *E. siraeum* and *O. valericigenes*. On one hand, *E. siraeum* is diminished in several conditions, including metabolically healthy obesity. Elevated levels of this bacterium and its metabolites have been associated with beneficial effects in gastric mucosa [89]. On the other hand, *O. valericigenes* has been identified as a key microbe that induces macrophage-associated inflammation in adipose target tissue and leads to adverse phenotypes of obesity and metabolic syndrome [90]. These results, in line with previous evidence, suggest the complexity of microbial interactions and that *E. siraeum* and *O. valericigenes* could play a role in metabolic health, and future studies to assess their clinical importance may be valuable.

Furthermore, the relative abundance of the genera *Coprococcus* was significantly enriched in the pre-dietary compared to the post-intervention overweight group (Table 7). An increase in this genus associated with preventive effects in T2D would be expected after dietary intervention. As mentioned above, this result may reflect a reorganization of the microbiota after the intervention, suggesting the importance of delving deeper into microbial taxa interactions [36,39].

These results suggest that, although the overall structure of the microbiota does not vary dramatically according to BMI, some taxa vary between normal weight samples and overweight subjects and respond differently to intervention. Our results align with previous evidence, recommending the consideration of BMI when designing precision nutrition strategies to optimize the preventive microbiota against T2D, as the response may depend on the BMI characteristics of each subject [91,92].

Precision nutrition can potentially optimize the T2D-preventive associated microbiota. However, several challenges should be addressed for implementing microbiome-based precision nutrition in clinical and public health settings. Creating a robust evidence base for bacterial biomarkers, reducing the costs of laboratory analyses, establishing food and microbiota relationships, and considering individual variability are essential to its effective application in preventing and managing T2D [16,17].

One of the main contributions of this study is the optimization of a high-resolution bacterial taxonomic classification to achieve a specific and reproducible microbial profile associated with T2D. Current scientific evidence suggests that bacterial taxonomic classification at the genus, species, and even subspecies level is essential to understanding the pathophysiological role of microbiota. Reaching an accurate taxonomic resolution depends mainly on the analyzed locus, the sequenced amplicon size, and the classification database selection. In this study, instead of 16S rRNA gene amplicons normally obtained from 200 to 400 bp, the entire gene (~1500 bp) is amplified and sequenced to improve taxonomic resolution [19].

In addition, we have selected the NCBI database for taxonomic classification because of its high resolution, down to the species and even subspecies level, which facilitates reliable identification of taxa potentially relevant for use in T2D prevention [20]. Unlike SILVA and RDP, which only classify down to the genus level, Greengenes allows for species-level classifications. A comparative analysis by Balvočiūtė and Huson indicated that, although SILVA, RDP, and Greengenes align well with NCBI, the correlation is not mutual. Therefore, NCBI taxonomy has been suggested as the standard framework for analyzing the 16S rRNA gene [20]. Moreover, the NCBI database is updated daily, uses over 150 sources, and is manually curated [93].

Nevertheless, taxonomic database selection is commonly determined by the pipeline used. Many studies that analyze T2D-associated microbiota employ SILVA, RDP, or Greengenes, limiting the taxonomic resolution of the previous results. It could be enriching to reanalyze them with the NCBI database to improve the scientific evidence regarding microbiota and T2D.

Moreover, the reduction in costs of microbiome-based precision nutrition is essential for facilitating its implementation in clinical practice. This article uses the sequencing platform MinION from Oxford Nanopore Technologies (ONT), which shows potential applicability in clinical practice due to its portability, low cost, and real-time sequencing compared with conventional sequencing technology [21,22]. However, the use of this technology in clinical settings associated with T2D still requires further validation to ensure accuracy and reproducibility.

Once target T2D preventive-associated bacteria have been identified (Table 1), the next step is to select prebiotics and related foods that theoretically favor their growth. This relationship can be established directly or indirectly, depending on whether the prebiotics contained in the food are prebiotics of the target bacteria and the amount of these prebiotics present in particular foods, which is a critical scientific challenge. This challenge is addressed by the bioinformatics food recommendation system, Phymofood (EP22382095), using an algorithm to quantify the influence of a group of foods on the state of the microbiota based on the established relationship. Thus, bioinformatics tools such as Phymofood (EP22382095) can personalize nutrition to promote optimal growth of T2D-preventive bacteria in the human gut microbiota. For example, Phymofood (EP22382095) has recently been explored to optimize the growth of CLA-producing gut microbiota bacteria and thereby maximize the anticancer effect of CLA in colorectal cancer [82].

Another key consideration in this article is that individual variability can affect the microbiota response to diet. Because of that, factors such as population group, age, blood sugar levels, and BMI that can influence the effects of precision nutrition interventions on microbiota composition are considered. Future studies should further explore the role of other factors, such as considering the genetic variants associated with interindividual variability in the target phenotypes [16,24]. In addition, for microbiome-based precision nutrition strategies to be sustainable in the long term, it is essential to consider individual health aspirations, food preferences, and barriers or facilitators of habit changes. This approach is a strength of these strategies, as it promotes acquiring long-term sustainable behavioral habits that are key to better and lasting health outcomes [94].

This study represents a proof of concept of the potential of applying microbiome-based precision nutrition for T2D prevention, but it has certain limitations. Although it advances towards its possible application in clinical practice, its results should be interpreted cautiously. The sample recruited is small and formed of healthy subjects from Spain with optimal glucose levels over 62 years of age, so the results may not fully represent the whole population. Further research with larger samples is needed to validate the results of this study. Moreover, research should be extended to populations with different genetic, lifestyle, and socioeconomic characteristics to explore the potential of microbiome-based precision nutrition for preventing T2D in different contexts. In addition, long-term longitudinal studies that analyze changes in the microbiota over time are needed to deepen the understanding of the role of specific bacteria and its interactions in T2D and establish possible causal relationships [16,17].

Another important point to address is that alterations in the gut microbiota of patients with prediabetes have been observed in some studies [95]. It is unclear whether there are differences in gut microbiota before the onset of prediabetes or T2D [96]. Prediabetes is an intermediate stage of dysglycemia commonly defined by impaired fasting glycemia (IFG) and impaired glucose tolerance (IGT), which identify partially overlapping groups [97,98]. The FG test is the primary screening method because of its reproducibility, low cost, and easy application. The OGTT has demonstrated high sensitivity in different populations, but its practical application is poorly reproducible, costly, and not well accepted by patients due to procedure requirements. For this reason, several guidelines suggest reserving the OGTT for particular situations. Moreover, the hemoglobin A1c (HbA1c) test is a widely recommended method for controlling previously diagnosed diabetes [25,99].

In this context, FG values < 110 mg/dL are considered normal in the Basque Health Service (Osakidetza) based on the CPG or <100 mg/dL according to the most restrictive ADA criteria [25]. However, it should be noted that even in the range of FG values < 100 mg/dL, 9.2% of the population may have IGT [100]. Within this subgroup with IGT, 75% do not progress to diabetes or can even revert to a normal glycemic state [101]. In addition, the risk of developing T2D decreases significantly with FG values below 95 mg/dL [102]. Because of that, our individuals were selected for normal FG values and values lower than 95 mg/dL (86 ± 5.5 mg/dL).

An additional important aspect to consider in future studies is the effect of metformin on gut microbiota. Recent studies suggest that metformin is a confounding factor in the study of T2D due to its ability to influence gut microbiota composition. Recent research suggests that some of the gut microbiota taxa previously associated with T2D may be explained by metformin treatment [103]. In addition, metformin use may mask the effects of dietary interventions on gut microbiota composition, and it should be analyzed in future studies [104].

Finally, to ensure equity in the implementation of microbiome-based precision nutrition in clinical and public health settings for T2D prevention and management, it is essential to balance investment in precision nutrition with the resources available for population-level public health strategies, ensuring that food education, nutrition policy, regulation, and legislation remain key elements in preventing T2D and associated complications [16,17].

Altogether, the results presented in this article open new research lines that could be useful for increasing our understanding of microbiota and T2D prevention and the promising applications of microbiome-based precision nutrition approaches as therapeutic tools for T2D.

## 5. Conclusions

Our review of the scientific evidence on bacteria with a potential preventive role against T2D identifies at least 48 bacteria with a significant association. After discarding those bacteria associated with detrimental health effects or inconsistent or insufficient evidence, 31 show strong evidence as potentially preventive against T2D. These findings support the growing scientific evidence suggesting that certain members of the human gut microbiota may be preventive against T2D.

The results of this study suggest that the microbiome-based precision nutrition tool could be a promising strategy to enhance the abundance of bacteria with a potential T2D-preventive role, thus promoting healthy population ageing. Specifically, we detected an enrichment of genera such as *Gemmiger* and species *G. formicilis and C. eutactus* in normal weight samples pre-dietary compared to post-dietary intervention. In contrast, genera such as *Butyrivibrio* and *Faecalibacterium* and species such as *E. crossota* and *F. prausnitzii* were enriched in the normal weight samples after the intervention. In addition, differences in the microbiota composition between normal weight and overweight samples were observed before and after the precision nutrition intervention.

This study addresses key challenges for implementing microbiome-based precision nutrition in clinical practice against T2D. However, the complete amplification of the 16S rRNA gene and the NCBI database are used to generate a specific, robust, and reproducible microbiota profile. In addition, the MinION sequencer is used due to its portability, cost-effectiveness, and real-time sequencing. The bioinformatics food recommendation system, Phymofood (EP22382095), is used for microbiota and food relationship identification. Finally, potential confounding factors must be considered to ensure that the effects of precision nutrition interventions on the microbiota are not masked. The use of this methodology has led to findings that are consistent with previous studies.

Microbiome-based precision nutrition interventions constitute strategies with great potential to prevent and manage T2D, opening the door to future approaches to healthy ageing. The results obtained in this study bring us closer to its future implementation in clinical practice. However, further research is needed to make progress in applying microbiome-based precision nutrition to larger and more diverse cohorts, as well as to describe an increasingly detailed profile of the gut microbiota bacteria associated with T2D and to elucidate the mechanisms involved.

## Figures and Tables

**Table 2:** Demographic and clinical data of samples.

Data	Normal Weight	Overweight
Age (mean, range)	67 ± 2	64 ± 1.3
BMI (kg/m^2^)	21 ± 1.4	27 ± 1.7
Female/male (%)	57.1/42.9	66.7/33.3
Blood sugar levels (mg/dL)	85 ± 5.8	87 ± 5.3
Personal food preferences (%)	28.6	33.3
Physical activity level (h/week)	9 ± 7.7	3 ± 3.2
Physical activity (%)		
Walking and yoga	14.3	50
Running, cycling, and swimming	57.1	50
Weights	28.6	0

**Table 3:** Bacterial taxa over-represented before dietary intervention compared to after in normal weight samples.

Taxa Name	Rank	Mean of Pre-Relative Abundance (%)	Mean of Post-Relative Abundance (%)	*p*-Value	Log 2 Fold Change	Occurrence in Pre (%)	Occurrence in Post (%)
*Gemmiger*	Genus	4.34	2.87	<10^−6^	0.6	100	100
*Gemmiger formicilis*	Species	4.34	2.87	<10^−6^	0.6	100	100
*Coprococcus eutactus*	Species	4.58	3.46	1.9 × 10^−5^	0.4	100	100

The taxonomic name refers to the current name in the NCBI Taxonomy Browser (https://www.ncbi.nlm.nih.gov/Taxonomy/Browser/wwwtax.cgi; accessed on 14 February 2025).

**Table 4:** Bacterial taxa under-represented before dietary intervention compared to after in normal weight samples.

Taxa Name	Rank	Mean of Pre-Relative Abundance (%)	Mean of Post-Relative Abundance (%)	*p*-Value	Log 2 Fold Change	Occurrence in Pre (%)	Occurrence in Post (%)
Bacillati	Kingdom	92.47	94.28	<10^−6^	−0.03	100	100
Bacillota	Phylum	42.02	42.93	1.5 × 10^−3^	−0.03	100	100
Clostridia	Class	40.11	41.71	<10^−6^	−0.06	100	100
Eubacteriales	Order	37.46	38.96	<10^−6^	−0.06	100	100
Lachnospiraceae	Family	14.68	16.85	<10^−6^	−0.2	100	100
*Butyrivibrio*	Genus	1.21	2.81	<10^−6^	−1.21	100	100
*Faecalibacterium*	Genus	3.11	4.06	7.2 × 10^−4^	−0.38	100	100
*Eshraghiella crossota*	Species	1.19	2.79	<10^−6^	−1.23	100	100
*Faecalibacterium prausnitzii*	Species	3.11	4.06	7.2 × 10^−4^	−0.38	100	100

The taxonomic name refers to the current name in the NCBI Taxonomy Browser (https://www.ncbi.nlm.nih.gov/Taxonomy/Browser/wwwtax.cgi; accessed on 14 February 2025).

**Table 5:** Over- and under-represented bacterial taxa in normal weight samples compared to overweight samples pre-dietary intervention.

Taxa Name	Rank	Mean of Pre-Normal Weight Relative Abundance (%)	Mean of Pre-Overweight Relative Abundance (%)	*p*-Value	Log 2 Fold Change	Occurrence in Pre-Normal Weight (%)	Occurrence in Pre-Overweight (%)
Bacillota	Phylum	42.02	37.41	<10^−6^	0.17	100	100
Clostridia	Class	40.11	36.36	<10^−6^	0.14	100	100
Eubacteriales	Order	37.46	33.95	<10^−6^	0.14	100	100
Bacteroidota	Phylum	4.27	12.5	<10^−6^	−1.55	100	100

The taxonomic name refers to the current name in the NCBI Taxonomy Browser (https://www.ncbi.nlm.nih.gov/Taxonomy/Browser/wwwtax.cgi; accessed on 14 February 2025).

**Table 6:** Over- and under-represented bacterial taxa in normal compared to overweight samples post-dietary intervention.

Taxa Name	Rank	Mean of Post-Normal Weight Relative Abundance (%)	Mean of Post-Overweight Relative Abundance (%)	*p*-Value	Log 2 Fold Change	Occurrence in Pre (%)	Occurrence in Post (%)
Lachnospiraceae	Family	16.85	13.86	<10^−6^	0.28	100	100
*Coprococcus*	Genus	5	2.8	<10^−6^	0.83	100	100
*Butyrivibrio*	Genus	2.81	1.52	2 × 10^−6^	0.88	100	100
*Eshraghiella crossota*	Species	2.79	1.51	2 × 10^−6^	0.88	100	100
*Coprococcus eutactus*	Species	3.46	1.98	<10^−6^	0.81	100	100
Oscillospiraceae	Family	14.65	16.94	<10^−6^	−0.21	100	100
*Oscillibacter*	Genus	2.67	3.88	1 × 10^−5^	−0.54	100	100
*Eubacterium siraeum*	Species	1.73	3.52	<10^−6^	−1.03	100	100
*Oscillibacter valericigenes*	Species	1.81	2.88	2 × 10^−4^	−0.67	100	100

The taxonomic name refers to the current name in the NCBI Taxonomy Browser (https://www.ncbi.nlm.nih.gov/Taxonomy/Browser/wwwtax.cgi; accessed on 14 February 2025).

**Table 7:** Over- and under-represented bacterial taxa before dietary intervention compared to after in overweight samples.

Taxa Name	Rank	Mean of Pre-Overweight Relative Abundance (%)	Mean of Post-Overweight Relative Abundance (%)	*p*-Value	Log 2 Fold Change	Occurrence in Pre-Overweight (%)	Occurrence in Post-Overweight (%)
Bacteroidota	Phylum	12.5	3.65	<10^−6^	1.78	100	100
*Coprococcus*	Genus	5.61	2.8	6.3 × 10^−4^	1	100	100
Bacillota	Phylum	37.41	42.91	<10^−6^	−0.20	100	100
Clostridia	Class	36.36	41.59	<10^−6^	−0.19	100	100
Eubacteriales	Order	33.95	38.83	<10^−6^	−0.19	100	100
Oscillospiraceae	Family	13.61	16.94	8 × 10^−6^	−0.32	100	100

The taxonomic name refers to the current name in the NCBI Taxonomy Browser (https://www.ncbi.nlm.nih.gov/Taxonomy/Browser/wwwtax.cgi; accessed on 14 February 2025).

## Data Availability

The original contributions presented in this study are included in the article/Supplementary Materials; further inquiries can be directed to the corresponding author.

## Supplementary Materials

The following supporting information can be downloaded at: https://www.mdpi.com/article/10.3390/nu17111877/s1, Supplementary Figure S1. α-diversity of normal weight samples compared to overweight samples pre-dietary intervention; Supplementary Figure S2. α-diversity of normal weight samples compared to overweight samples post-dietary intervention; Supplementary Table S1. ANOVA and PERMANOVA test for β-diversity of microbiota in normal weight samples compared to overweight samples pre- and post-dietary intervention. Supplementary Figure S3. α-diversity of pre- and post-dietary intervention from overweight samples; Supplementary Figure S4. β-diversity of pre-dietary compared to post-dietary intervention from overweight samples. The principal coordinates analysis (PCoA) plots were based on two metrics: Bray–Curtis dissimilarity and the Jaccard index (ellipses represent 95% confidence intervals).

## Abbreviations

The following abbreviations are used in this manuscript:

ADA	American Diabetes Association
BMI	body mass index
BCAAs	branched-chain amino acids
CPG	Clinical Practice Guidelines
FG	fasting glucose
HbA1c	hemoglobin A1c
IDF	International Diabetes Federation
IFG	impaired fasting glycemia
IGT	impaired glucose tolerance
OGTT	oral glucose tolerance test
SCFAs	short-chain fatty acids
T2D	type 2 diabetes
WHO	World Health Organization
16S rRNA	16S ribosomal RNA
