# Supplementary material for: In Search of Healthy Ageing: A Microbiome-Based Precision Nutrition Approach for Type 2 Diabetes Prevention
Source: Nutrients. 2025 May 30;17(11):1877. doi: 10.3390/nu17111877 (PMC12158179; doi:10.3390/nu17111877)
Supplement: Supplementary file 1 [file nutrients-17-01877-s001.zip › Supplementary Table S1.pdf]

Supplementary Table S1. ANOVA and PERMANOVA test for β-diversity of microbiota in normal weight samples compared to overweight samples pre- and post-dietary intervention.

	**β-diversity**			
	Pre-intervention Normal weight vs. overweight		Post-intervention Normal weight vs. overweight	
Metric	ANOSIM	PERMANOVA	ANOSIM	PERMANOVA
Bray-Curtis	R=0.0265	F=1.018	R=−0.0674	F=0.8425
	p=0.3346	*p=0.4218*	*p=0.713*	*p=0.5552*
Jaccard	R=0.0714	F=1.196	R= 0.0159	F=1.006
	p=0.1646	*p=0.144*	*p=0.3626*	*p=0.4125*
