# Supplementary figures and images for: In Search of Healthy Ageing: A Microbiome-Based Precision Nutrition Approach for Type 2 Diabetes Prevention
Source: Nutrients. 2025 May 30;17(11):1877. doi: 10.3390/nu17111877 (PMC12158179; doi:10.3390/nu17111877)

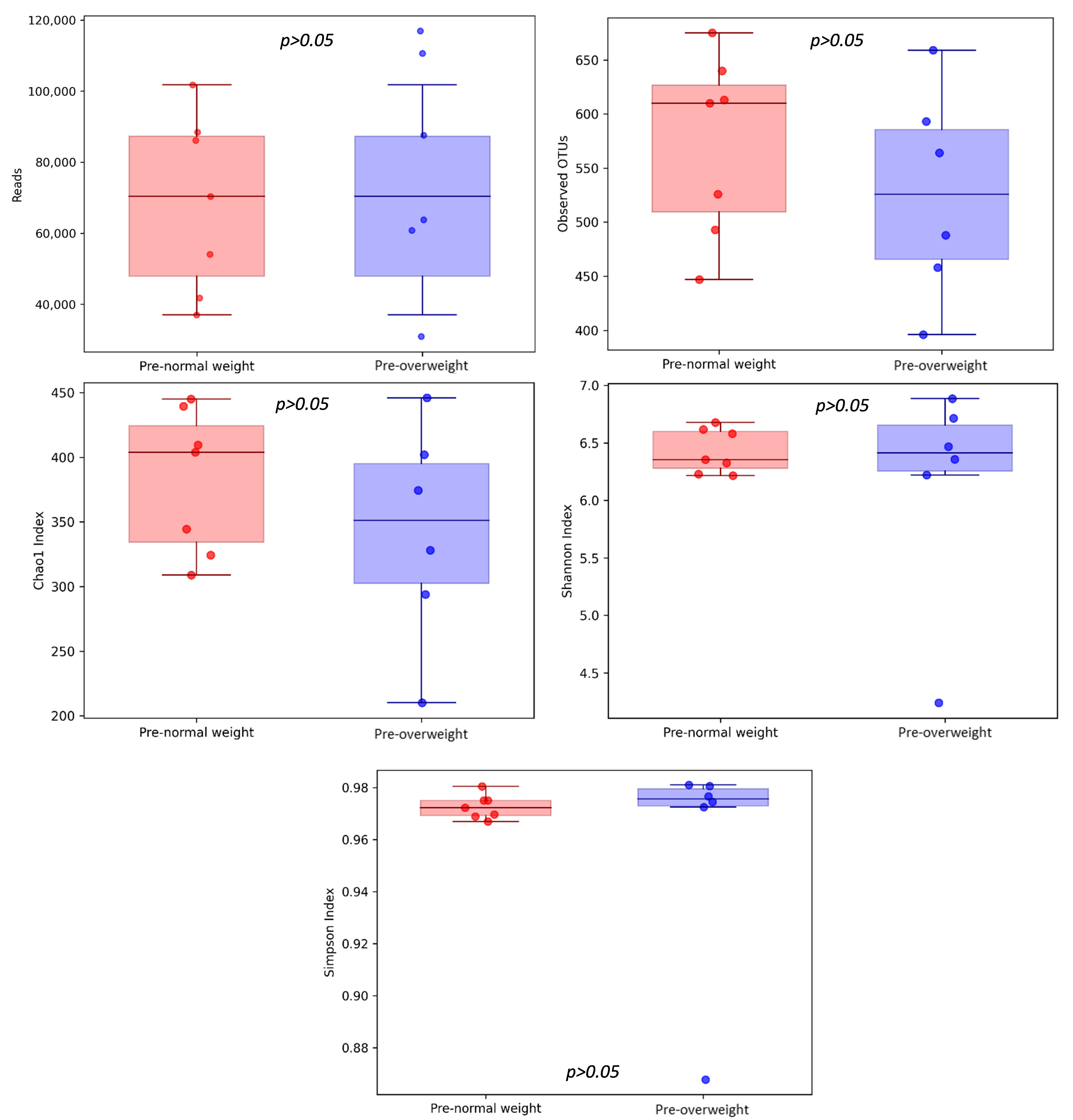

Supplement: Supplementary file 1 [file nutrients-17-01877-s001.zip › SupplementaryFigureS1.png]

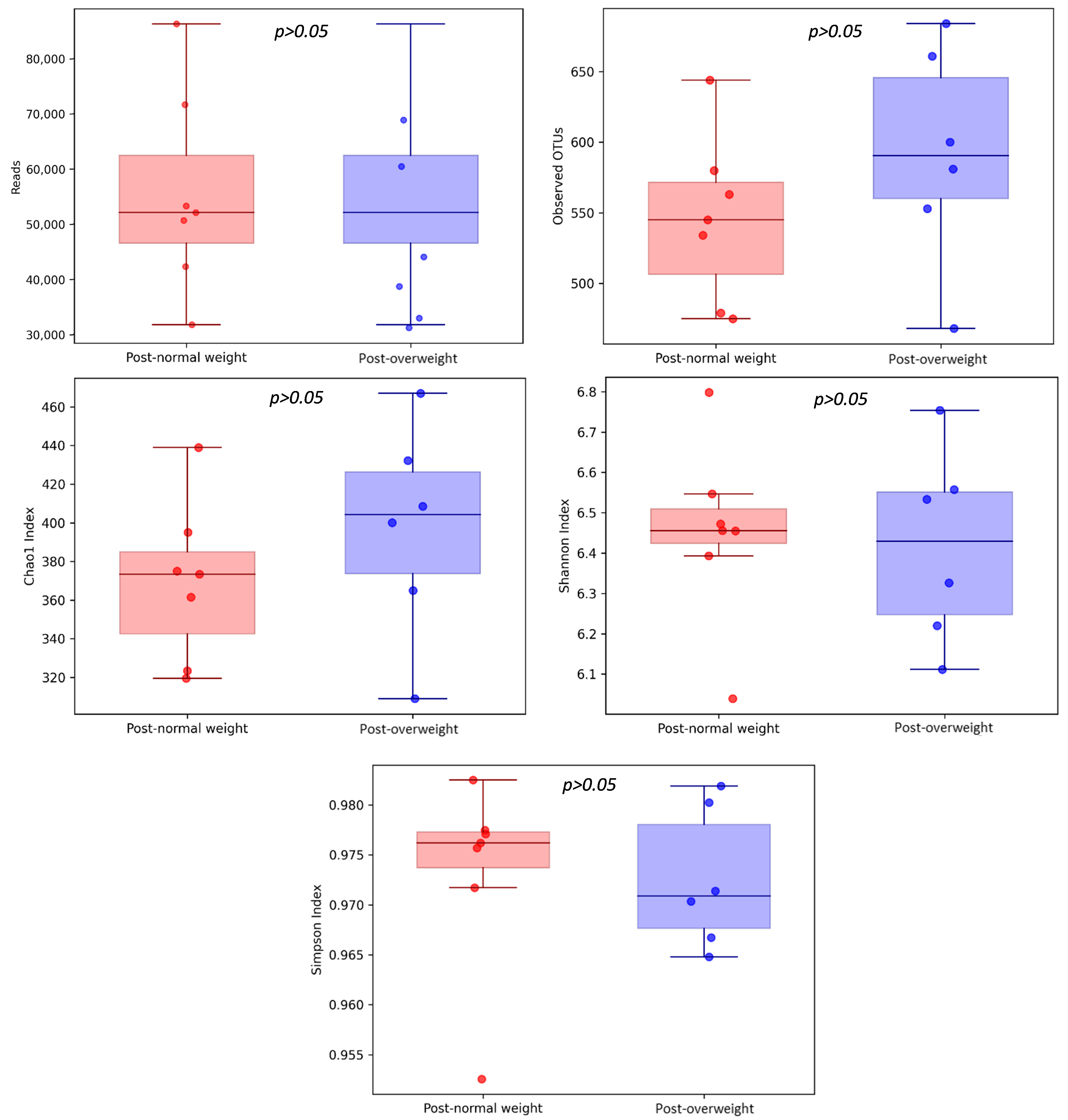

Supplement: Supplementary file 1 [file nutrients-17-01877-s001.zip › SupplementaryFigureS2.png]

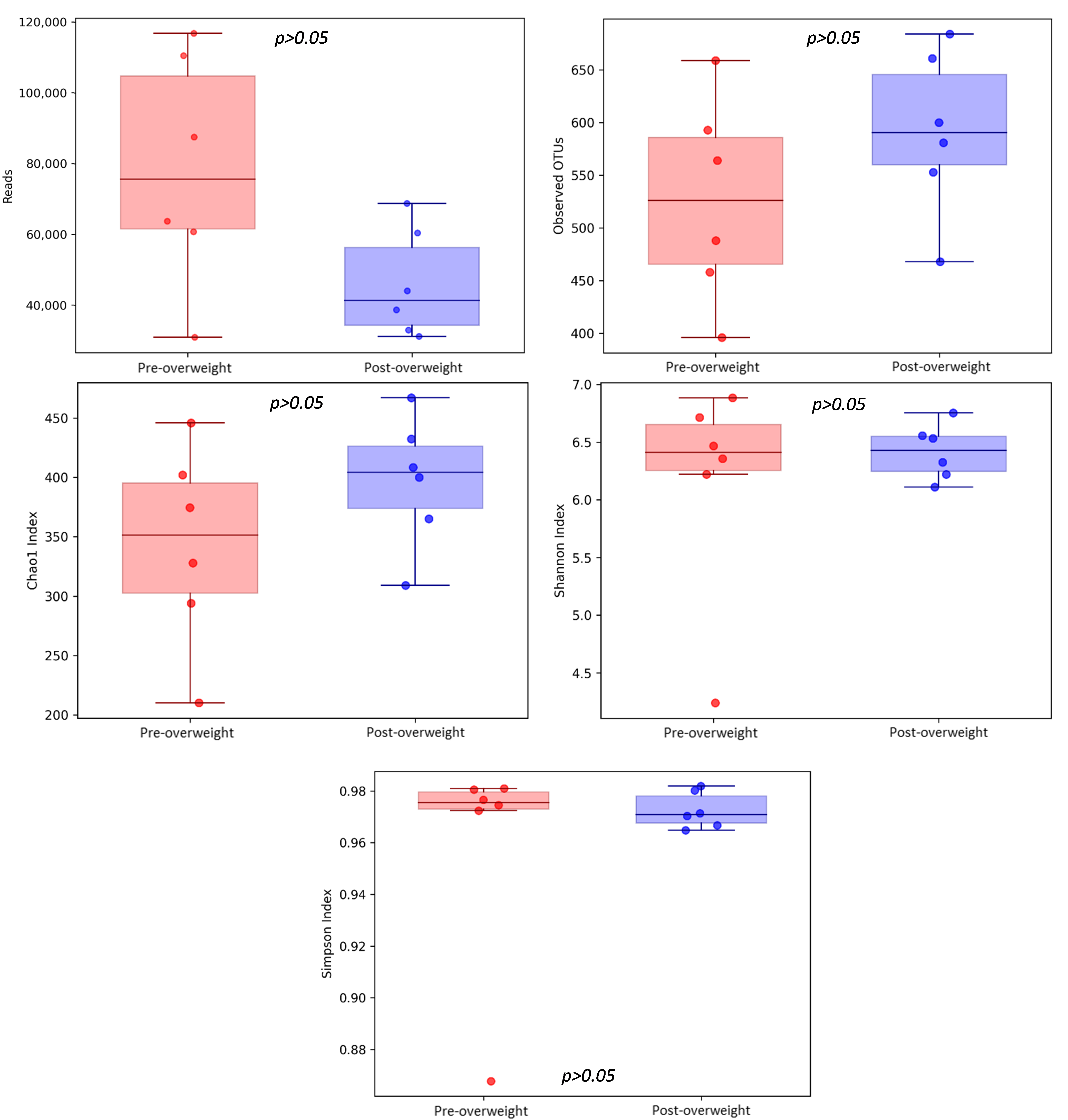

Supplement: Supplementary file 1 [file nutrients-17-01877-s001.zip › SupplementaryFigureS3.png]

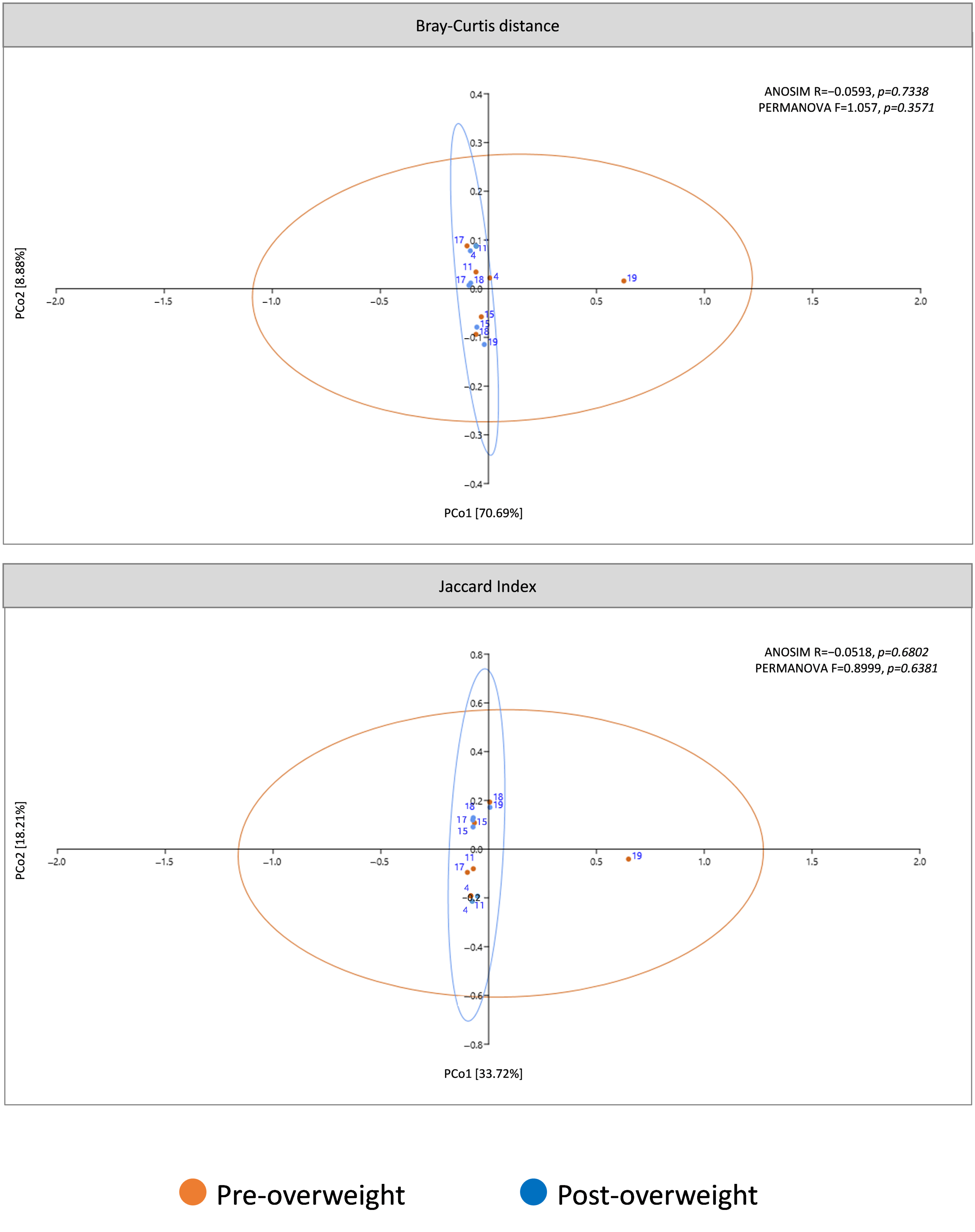

Supplement: Supplementary file 1 [file nutrients-17-01877-s001.zip › SupplementaryFigureS4_quality.png]
